# Implementation of a complex intervention for hip fracture care: a protocol for an embedded mixed-methods process evaluation of a multicentre cluster randomised trial in India, Nepal, the Philippines, Thailand and Vietnam

**DOI:** 10.1136/bmjopen-2026-124609

**Published:** 2026-10-20

**Authors:** Elizabeth Armstrong, Rebecca Ivers, Thuy Anh Bui, Paphon Sa-Ngasoongsong, Saowalak Tongta, Maneemon Phrommai, Watcharida Pinyo, Dipendra Pandey, Pranab Dahal, Situ Manandhar, Reshika Shrestha, Sunil Kumar Joshi, Jagnoor Jagnoor, Deepti Beri, Medhavi Gupta, Vijay Sharma, Quang La, Cuong Pham, Kiko Cortez, Irewin Tabu, Matthew L Costa, Anna Palagyi, HIPCARE Investigators, Matthew L Costa

**Affiliations:** 1 School of Population Health, UNSW Sydney, Sydney, New South Wales, Australia; 2 Department of Orthopedics, Mahidol University Faculty of Medicine, Ramathibodi Hospital, Bangkok, Thailand; 3 Department of Orthopedics and Trauma Surgery, National Trauma Centre, National Academy of Medical Sciences, Kathmandu, Central Development Region, Nepal; 4 Faculty of Health and Life Sciences, Department of Health and Caring Sciences, Linnaeus University, Kalmar, Kalmar County, Sweden; 5 Kathmandu Medical College, Kathmandu, Central Development Region, Nepal; 6 Lubhu Primary Health Care Center, Lalitpur, Nepal; 7 Injury Division, The George Institute for Global Health India, New Delhi, India; 8 Injury Division, The George Institute for Global Health, Randwick, New South Wales, Australia; 9 Department of Orthopaedics, All India Institute of Medical Sciences Jai Prakash Narayan Apex Trauma Center, New Delhi, India; 10 Hanoi University of Public Health, Hanoi, Vietnam; 11 Center for Injury Policy and Prevention Research, Hanoi University of Public Health, Hanoi, Vietnam; 12 Department of Orthopaedics, University of the Philippines Manila, Manila, NCR, Philippines; 13 The Kadoorie Institute for Trauma, Emergency and Critical Care, Nuffield Department of Orthopaedics, Rheumatology and Musculoskeletal Science, University of Oxford, Oxford, UK; 14 The George Institute for Global Health, Sydney, New South Wales, Australia; 15 Nuffield Department of Orthopaedics, Rheumatology and Musculoskeletal Science, University of Oxford, Oxford, UK

**Keywords:** Hip, Quality Improvement, QUALITATIVE RESEARCH, Delivery of Health Care, Integrated, Fractures, Bone, GERIATRIC MEDICINE

## Abstract

**Introduction:**

The HIPCARE Study is a cluster randomised trial to determine whether the introduction of a multidisciplinary model of hip fracture care in five low- and middle-income countries (LMICs) in Asia can improve quality of life and reduce healthcare costs for older people undergoing surgery. An embedded process evaluation will provide detailed information on the contextual factors influencing the implementation of HIPCARE to explain what works, for whom and why. This paper describes the protocol for the HIPCARE process evaluation.

**Methods and analysis:**

A mixed-methods process evaluation will be embedded in the HIPCARE trial, informed by two theoretical frameworks: the realist framework (context, mechanism, outcome) and the reach, effectiveness, adoption, implementation and maintenance framework. Two intervention hospitals from each of the five implementation countries will be selected as sites for the process evaluation (10 sites in total). For each site, quantitative data from the HIPCARE implementation dashboard and main trial outcomes will be used to examine relationships between implementation processes and patient outcomes, care processes and intervention delivery. Qualitative data will comprise ~100 interviews or mini focus group discussions with multidisciplinary team members, administrators and trial staff at site setup and recruitment midpoint and endpoint, alongside patient and caregiver focus groups (two per site, 6–8 participants each) at 60- and 120-days post-surgery. Quantitative and qualitative data will be triangulated through an explanatory integration approach, in which implementation process measures and outcomes are interpreted alongside patient and provider experiences and contextual insights to identify convergent and divergent findings. This triangulation will enable identification of mechanisms linking intervention delivery to outcomes and clarify how contextual factors shape adoption, implementation and sustainability across settings.

**Ethics and dissemination:**

The study has been approved by the Oxford Tropical Research Ethics Committee (OxTREC Reference: 520–23) and ethics committees in each participating country. Findings will be shared with policy makers, healthcare workers and community representatives through social media, webpages, peer-reviewed publications, presentations and printed resources.

**Trial registration number:**

ISRCTN23534273.

**STRENGTHS AND LIMITATIONS OF THIS STUDY**

- Asia is a region with rapidly ageing populations, which will have implications for government and health system policies to ensure they are responsive to the needs of older people.
- The process evaluation will be conducted in half of the intervention hospitals, and this may limit representativeness.
- Purposive sampling of the process evaluation hospitals has the potential to enhance the capture of implementation experiences by ensuring diverse descriptions of organisation and contextual characteristics and influences.
- The process evaluation uses two theoretical frameworks to ensure a robust approach into the contextual factors influencing the delivery, sustainability and potential scale-up of team-based multidisciplinary care for older people who fracture their hip in India, Nepal, the Philippines, Thailand or Vietnam.
- The process evaluation is strengthened by local leadership, local researchers and consumer involvement bringing depth of knowledge of the contextual setting in which the intervention is to be evaluated.

## Introduction

 Older people frequently sustain fractures after falls from a standing height, as their bones are weakened by osteoporosis. Hip fracture is the most common ‘fragility’ fracture requiring treatment in hospital. These fragility fractures have serious consequences, with up to 25% of patients with hip fracture not surviving 1 year after injury^1 2^ and survivors experiencing a reduction in their health-related quality of life comparable to having a stroke.^3^

The number of patients with fragility fractures is increasing dramatically in low- and middle-income countries (LMICs) due to the changing demographics of their populations.^4^ Modelled data from nine Asian LMICs indicate a predicted twofold increase in the number of hip fractures, from 1 124 060 in 2018 to 2 563 488 in 2050,^5^ with associated healthcare costs increasing from US$9.5 to $15 billion.^4^ Without improvements in processes and outcomes, health and social care systems in these settings are likely to be challenged to provide equitable access to hip fracture care that meets the needs of older people with this injury.

In high-income countries, it has been shown that implementing a programme of multidisciplinary teamwork, where nurses, therapists, surgeons and medical specialists work together as a team (joint responsibilities and defined accountabilities) reduces time to surgery, length of stay, costs of care and mortality following hip fracture.^6 7^ However, current available evidence from LMICs in Asia indicates that a multidisciplinary orthogeriatric approach to hip fracture care is rare.^8^ In the Asia-Pacific region, Tabu *et al* showed that, in 2020–2021, the median time to surgery in Thailand was 3–4 days compared with 5 days or more in the Philippines and Nepal.^8^ Similarly, while specialist orthogeriatricians were available in many centres in Vietnam and India, much of the medical care for older people with hip fracture in the Philippines and Thailand was provided by rehabilitation medicine or acute medicine specialists. Furthermore, while nursing and physiotherapy staff were available in nearly all centres, they were not usually empowered to mobilise hip fracture patients with unrestricted weight-bearing on the first day following surgery.^8^ Another study from China—an upper-middle income country—highlighted long delays to surgery and lack of availability of geriatrician assessment within the hip fracture care pathway.^9^ However, the same hospital subsequently showed that a multidisciplinary care intervention was cost-effective and significantly improved the timeliness of surgery, assessment of osteoporosis and survival at 1 year.^10 11^

### The HIPCARE study

The HIPCARE study is a multicentre, interventional cluster randomised controlled trial of a multidisciplinary care package for hip fracture care implemented in five countries in Asia: India, Nepal, the Philippines, Thailand and Vietnam (https://www.isrctn.com/ISRCTN23534273). The trial includes an embedded process evaluation to provide detailed information on the contextual factors influencing success or failure of the proposed intervention in each setting. The multidisciplinary care package—engaging orthopaedic surgeons, physicians with a special interest in the care of older persons and allied health staff—will address the timeliness of three key elements of hip fracture care: 1) surgery to fix the fracture within 36 hours of admission; 2) assessment by a doctor specialised in the care of older people within 72 hours of admission and 3) standing and walking with unrestricted weight-bearing within 24 hours of surgery.

In each of the 5 countries, 8 hospitals (40 hospitals in total) that each treats 200+ patients with hip fractures annually will be recruited, with four hospitals randomly allocated to the intervention group and the remaining four hospitals acting as controls. Each hospital will target recruitment of at least 200 patient participants during the planned recruitment period from November 2024 to 31 July 2026. Data collection and data quality checks for the main trial will continue until the end of December 2026. HIPCARE intervention hospitals will receive initial training and support to optimise the care pathway for patients with hip fracture. Each intervention hospital will appoint a multidisciplinary care ‘champion’ who will chair a monthly multidisciplinary care meeting to review data regarding time to surgery, time to unrestricted weight-bearing and mobilisation and time to comprehensive physician assessment. The data will be compiled via a live electronic dashboard. The multidisciplinary team will be empowered to adopt the optimised approach for all patients presenting to the hospital during the trial period. Patient participants are eligible for inclusion if they are aged 60 years or over and admitted to an intervention hospital for surgery for a hip fracture and available for follow-up at 120-days after surgery. For patients with hip fracture presenting to control hospitals, there will be no change to the usual treatment pathway. In both control and HIPCARE intervention hospitals, only one treatment pathway will be available.

### The HIPCARE process evaluation

The HIPCARE process evaluation aims to facilitate the sustainability and scale-up of the intervention by understanding the factors influencing implementation of HIPCARE at the participating trial sites. This process evaluation extends earlier research exploring clinician and administrator perspectives of hip fracture care in LMICs, which showed a mismatch between the clinical needs of older people and the health system’s ability to meet those needs.^12^ The process evaluation will explore the multidisciplinary care package, the online dashboard to collate and present data on the key metrics (ie, time to surgery, physician assessment and mobilisation) and the formation of a hospital-based multidisciplinary team to regularly review the collated data. Implementation of the HIPCARE intervention package will require adjustment of hospital systems and existing processes and resourcing. The nature of these adjustments is likely to be unique to each implementation setting and will be dependent on factors such as hospital governance, resource availability and the attitudes and acceptance of healthcare workers.

To understand and explain HIPCARE outcomes, it is necessary to examine the contextual factors that may influence HIPCARE implementation, that is, what it was about the HIPCARE intervention that worked (or did not work) in the unique participating sites, for whom did the HIPCARE intervention work (and did not work) and why was this the case.^13^ The embedded process evaluation is designed to identify those contextual factors that may both undermine or enhance the implementation of the HIPCARE intervention and therefore its impact and sustainability. The evaluation will also highlight how these factors may vary between participating hospitals within countries and between the five participating countries.^14^ Primary and secondary objectives of the process evaluation are:

- Primary objective: To evaluate processes underpinning implementation of the HIPCARE intervention and explore end-users’ perceptions of it, to give a deeper understanding of its effects.
- Secondary objective: To provide recommendations for intervention scale-up and transferability.

## Methods and analysis

A mixed-methods process evaluation, informed by the UK Medical Research Council guidance,^13 14^ will be employed to provide detailed information on the contextual factors influencing success or failure of the HIPCARE intervention in each setting. Quantitative data from HIPCARE intervention hospitals will be used to examine whether variation in outcomes aligns with factors influencing implementation, thereby supporting interpretation of why and how the study outcomes occurred. A summary of the quantitative data to be used in the process evaluation is provided in table 1.

**Table 1:** HIPCARE process evaluation quantitative data variables

Data	Source
Patient characteristics (age, sex, rurality)	Main trial analysis
HRQoL @ 120 days post-surgery (age, sex, rurality)	Main trial analysis
% survived to 120 days post-surgery (age, sex, rurality)	Main trial analysis
% returned to pre-fracture living location at 120 days postop (age, sex, rurality)	Main trial analysis
% experiencing complications up to 120 days post-surgery (age, sex, rurality)	Main trial analysis
Cost-effectiveness	Main trial analysis
% operated within 36 hours, by age and sex	Dashboard
% assessed within 72 hours, by age and sex	Dashboard
% walking within 24 hours of surgery, by age and sex	Dashboard
Number and frequency of multidisciplinary team meetings	Dashboard

.HRQoL, health-related quality of life.

In addition, qualitative research will be conducted to understand context, with data collection timepoints, data sources and data collection methods illustrated in figure 1. Timing of process evaluation data collection in each country will be influenced by the date of the first patient recruitment to the main trial. Following the end of main trial recruitment on 31 July 2026, participant recruitment, data collection and data quality checks for the process evaluation will continue until December 2026 for all five countries. Ten of the 20 intervention hospitals will be selected to ensure diversity across characteristics likely to influence implementation of team-based care, including hospital size, geographic location and service profile. This approach is designed to obtain a broad range of implementation contexts within each country and allow the capture of diverse implementation experiences. From a diverse, representative sample of 10 hospitals (2 HIPCARE intervention hospitals from each of the 5 trial countries), approximately 100 interviews or mini focus groups will be conducted with the trial coordinators, country leads, intervention champions, hospital or health service administrators and members of the multidisciplinary team. Data collection will occur at study set-up, midpoint and endpoint, aligned with the commencement of main trial recruitment (site set-up) and completion of recruitment on 31 July 2026 (endpoint). Data collection will be structured to explore the contextual facilitators and barriers to implementation, the adoption of and adaptations to the intervention and its perceived impact. Site-specific data from meeting minutes of the hospital-based multidisciplinary team (MDT) will also be used to inform the implementation context. To assess patient and caregiver acceptability and perceived effectiveness, two focus groups per hospital, each with 6–8 patient and caregiver participants, will be conducted at approximately 60- and 120-days post-surgery.

**Figure 1:**
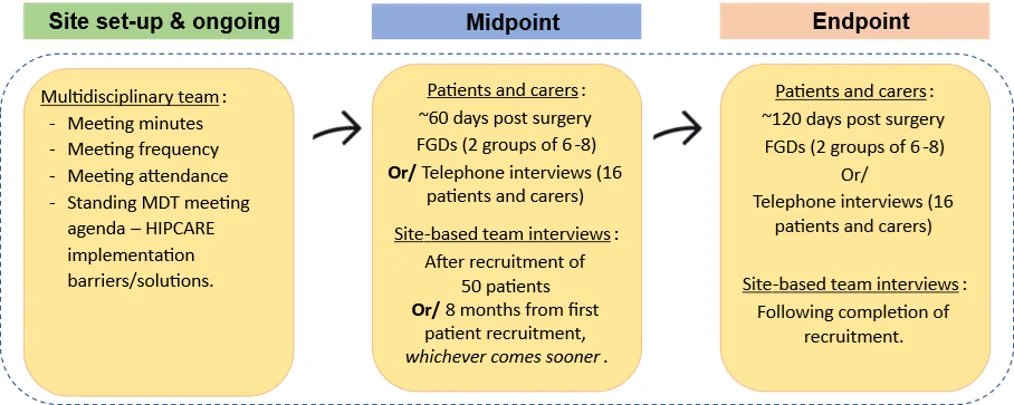
Timeline of qualitative data collection in the HIPCARE process evaluation. MDT, multidisciplinary team.

In each country of HIPCARE implementation, an academic or academic-affiliated institution will act as the trial coordinating centre, with the country lead investigator overseeing the study activities and a trial coordinator responsible for facilitating and managing the operational aspects of the study. For the process evaluation, each trial coordinating centre will appoint a local qualitative researcher to coordinate data collection at the relevant timepoints. An evaluation team based in Sydney, Australia, will provide support to the qualitative researcher(s) and the trial coordinator in each country. Prior to patient recruitment commencing for the main trial, the evaluation team will provide resources and training to staff at each coordinating centre via scheduled virtual meetings. Support will continue to be provided to each country team, as required, during the recruitment phase of the main trial.

### Process evaluation analysis framework

The approach to the process evaluation has been informed by two theoretical frameworks. First, the reach, effectiveness, adoption, implementation and maintenance framework (RE-AIM)^15^ provides a structural approach to examining the proportion and representativeness of the participants involved in the intervention, the impact of the intervention, the adoption and acceptability of the intervention, the implementation of the intervention and how the intervention may be institutionalised within the health system. Second, the realist framework (context, mechanism, outcomes)^16^ will be used in the process evaluation to provide a theoretical basis for identifying potential causal mechanisms for how the HIPCARE intervention works, for whom and under what contexts, therefore fostering uptake of research-based knowledge into practice (see figure 2).

**Figure 2:**
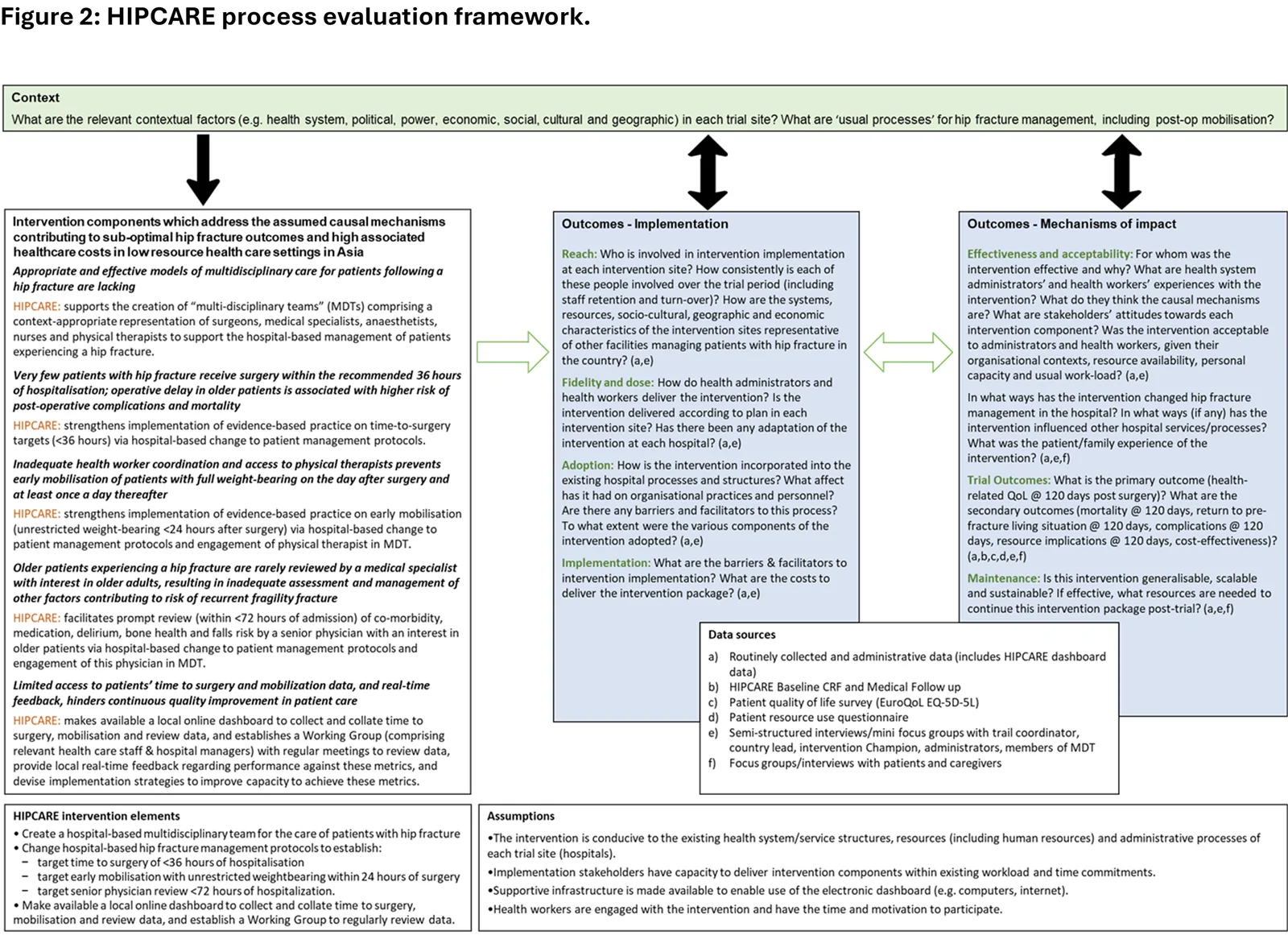
HIPCARE process evaluation framework. MDT, multidisciplinary team; CRF, Case Report Form.

The HIPCARE process evaluation will guide the assessment of the contextual factors within which the intervention is implemented to enable documentation of the barriers, facilitators, research, adoption and costs associated with the implementation of HIPCARE. The process evaluation will also inform the intervention’s mechanisms of impact, including aspects of effectiveness, scalability and sustainability. The frameworks will aid the process evaluation analysis by enabling the triangulating of quantitative process measures with qualitative data from key stakeholders involved in the intervention implementation. It will also provide a structured template to consolidate the findings, while remaining flexible and open to refinement as the evaluation progresses.

### Data management and analysis

All process evaluation data collected during the HIPCARE Study will be handled and stored in accordance with local and national data protection legislation. Identifiable information, including contact details and consent documentation, will only be accessible to research staff from participating centres in each country. Where any data is transferred to other institutions for analysis, it will be pseudonymised before transfer from the host country, using existing research information technology. Any reports and other outputs of the study will not directly or indirectly identify individual participants.

The qualitative data will be transcribed, translated and analysed with input from each HIPCARE country lead investigator and qualitative researcher to ensure in-depth knowledge and appropriate interpretation of local context. Qualitative data will be analysed using a mix of top-down (deductive) and bottom-up (inductive) coding. The first set of coding will be top-down, guided by the Realist framework in which parts of the data are identified as context, mechanism or outcome. Within each category, further bottom-up coding will identify themes that expand these constructs. All transcripts will be independently coded by at least two researchers. Regular meetings will be held to compare coding, discuss interpretations and refine the coding framework. Discrepancies in coding will be resolved through discussion and consensus with the involvement of researchers from each of the participating countries during the analysis process. For interviews conducted in languages other than English, audio files will be transcribed into the local language prior to translation into English-language by qualified translators or bilingual researchers. English-language transcripts will be reviewed for accuracy and original-language transcripts will be consulted during analysis when clarification of meaning is required.

### Ethics approvals and consent to participate

The study, including the embedded process evaluation, was approved by the Oxford Tropical Research Ethics Committee on 13 July 2023 (OxTREC Reference: 520–23), the George Institute Ethics Committee, dated 27 September 2023 (TGIEC Reference: 22/2023), Nepal Health Research Council, dated 8 August 2024 (NHRC Reference: 371_2024), the Philippines Single Joint Research Ethics Board, dated 11 December 2023 (SJREB Reference: 2023–93), the National Research Council of Thailand Central Research Ethics Committee, dated 25 June 2024 (CREC Reference: 047/67BT-BIO8) and Hanoi University of Public Health Ethical Review Board for Biomedical Research, dated 22 January 2025 (Reference: 025–023/DD-YTCC). The study was ratified by UNSW Research Ethics and Compliance (iRECS8013). Signed informed consent for the process evaluation will be obtained from all participants contributing data. If a participant is unable to read or if a legally acceptable representative is unable to read, an impartial witness would be present during the entire informed consent discussion and will attest informed consent.

### Patient and public involvement

The views and perspectives of patients and caregivers from Asia and the UK have informed all aspects of the main HIPCARE trial, including the embedded process evaluation, the study design and oversight. There will be ongoing engagement with patients and caregivers throughout the process evaluation via the HIPCARE Community Engagement and Involvement Groups in each country.

### Dissemination

The findings of the HIPCARE trial will be shared with three key groups: policy makers, healthcare workers and community engagement and involvement representatives. The findings will be disseminated through plain language summaries specific to each country with distribution through social media channels, webpages and printed hard copies. Findings will also be shared by the investigators and country teams through scientific, peer-reviewed publications and presentations at global events of the Fragility Fracture Network and other organisations.

## Discussion

This embedded process evaluation of the HIPCARE c-RCT will be the first to explore the implementation of a complex intervention for the care of older people with a hip fracture in LMICs in Asia. The results will provide an important contribution by exploring the implementation of key elements of timely hip fracture care. Improved understanding of the applicability and generalisability of early surgery, early physician assessment and early, unrestricted walking after hip fracture surgery will help to inform strategies for sustainability after the research study has been completed.

The process evaluation may also help to illuminate possible reasons for the findings of recent systematic reviews that identified variation in mortality after hip fracture in many countries and global regions, including the Asia-Pacific.^1 2 17^ The most recent review^17^ reported a meta-analysis of mortality from 14 countries, seven of which were LMICs including four of the five countries participating in HIPCARE. All seven LMICs showed lower mortality estimates than two high-income countries, Australia and New Zealand, for in-hospital and 30-day mortality, and six of seven LMICs showed lower 1 year mortality than both Australia and New Zealand. Differences in patient case-mix, healthcare access and admission may contribute to the observed variation in survival following hip fracture. Understanding these variations across the diverse trial settings through the process evaluation may provide meaningful insights into the factors influencing the delivery (and subsequent outcomes) of hip fracture care. In this way, the process evaluation may contribute to the interpretation of country-level differences in observed patient outcomes.

A strength of the process evaluation will be the application of two theoretical frameworks to understand what it was about the HIPCARE intervention that worked or did not work, for whom and why was this the case. The RE-AIM Framework^15^ will apply a standardised approach to a comprehensive inquiry into the implementation of the HIPCARE intervention within and between the five participating countries. A challenge to the use of the RE-AIM will be the intervention itself, which is based on the provision of multidisciplinary care to achieve three specific elements of care: early surgery, medical assessment and postoperative walking. The multidisciplinary care may vary from hospital to hospital depending, for example, on the type of physician providing early medical assessment. Teasing out the differences of an intervention defined by its achievement of the three elements will be important for sustainability and potential scale-up. This challenge will be mitigated by using a Realist framework^16^ to explore the context, mechanism and outcomes of the intervention’s implementation. Understanding how HIPCARE is implemented in diverse settings will provide real-world information on how the HIPCARE intervention will produce the identified outcomes.

We recognise there may be limitations of the proposed approach, specifically related to the contextual assumptions made regarding the implementation of HIPCARE. The intervention may not be supported by the existing structures of the health system or service or supportive infrastructure such as internet stability may be lacking or intermittently available, impacting the collection and use of data for feedback. There may also be challenges to data collection from patients and caregiver participants due to logistical barriers to attend post-surgical review. We have attempted to anticipate some limitations on qualitative data collection and have taken a pragmatic approach by accommodating either face-to-face or telephone/virtual modes of data collection and the use of either focus group discussion or semi-structured interview, depending on the preference of patient or caregiver participants.

With interview-based research there is the potential for bias due to cross-cultural differences in language, comprehension, emotions, experiences, professional norms, organisational cultures or attitudes towards team-based care.^18^ These differences may affect interpretation and comparability of the findings. There may also be some loss of nuance and contextual meaning during translation from local languages into English. Another limitation may be the potential for social desirability bias,^19^ whereby implementation champions, hospital leaders and other stakeholders involved in intervention delivery provide socially desirable responses, potentially leading to more favourable descriptions of implementation processes and experiences. In addition, distinguishing between intervention fidelity and contextually appropriate adaptations may be challenging, particularly in complex health system settings where implementation strategies are intentionally tailored to local circumstances. Finally, as only a subset of intervention hospitals will participate in the process evaluation, experiences and contexts may not fully reflect non-participating sites. This may lead to variation in data generation and interpretation and may limit the transferability of identified barriers, enablers and adaptations; however, the process evaluation is designed to understand implementation within diverse, real-world settings and this may be considered a focus of the evaluation rather than a limitation of the proposed approach.

Notwithstanding the effectiveness of the HIPCARE intervention, the process evaluation will provide meaningful insights into the contextual mechanisms contributing to the main trial outcomes. It will generate in-depth evidence of the implementation of the HIPCARE intervention across diverse LMIC settings and elucidate how contextual factors shape adoption, fidelity and outcomes. These findings will have important implications for the future provision of hip fracture care in LMICs and will also inform the future directions of related research in these settings. Should the HIPCARE intervention be successful in reducing the costs associated with hip fracture care and also improve patient quality of life, then the process evaluation will be able to guide optimisation and scale-up of multidisciplinary hip fracture care within HIPCARE implementation countries and inform transferability to resource-constrained health systems elsewhere.

### Trial status

This manuscript reports HIPCARE protocol V.2, 24 March 2026. The HIPCARE Study commenced in November 2022 and is expected to run until March 2027. Patient recruitment to the main trial commenced in December 2024 and recruitment is scheduled to finish on 31 July 2026.
