# Supplementary material for: Implementation of a complex intervention for hip fracture care: a protocol for an embedded mixed-methods process evaluation of a multicentre cluster randomised trial in India, Nepal, the Philippines, Thailand and Vietnam
Source: BMJ Open. 2026 Oct 20;16(10):e124609. doi: 10.1136/bmjopen-2026-124609 (PMC13684186; doi:10.1136/bmjopen-2026-124609)
Supplement: Supplementary file 1 [file bmjopen-2026-124609.reviewer_comments.pdf]

PEER REVIEW HISTORY

BMJ Open publishes all reviews undertaken for accepted manuscripts. Reviewers are asked to complete a checklist review form and are provided with free text boxes to elaborate on their assessment. These free text comments are reproduced below.

ARTICLE DETAILS

Title (Provisional)

Implementation of a complex intervention for hip fracture care: A protocol for an embedded mixed-methods process evaluation of a multicentre cluster randomised trial in India, Nepal, the Philippines, Thailand and Vietnam

Authors

Armstrong, Elizabeth; Ivers, Rebecca; Bui, Thuy Anh; Sa-ngasoongsong, Paphon; Tongta, Saowalak; Phrommai, Maneemon; Pinyo, Watcharida; Pandey, Dipendra; Dahal, Pranab; Manandhar, Situ; Shrestha, Reshika; Joshi, Sunil Kumar; Jagnoor, Jagnoor; Beri, Deepti; Gupta, Medhavi; Sharma, Vijay; La, Quang; Pham, Cuong; Cortez, Kiko; Tabu, Irewin; Costa, Matthew L; Palagyi, Anna; Investigators, HIPCARE

VERSION 1 - REVIEW

**Reviewer**	**1**
**Name**	**Morrell, Aidan**
**Affiliation**	**Oregon Health & Science University, Orthopaedics**
**Date**	**13-Sep-2026**
**COI**	**None**

Minor revision

Summary

This is a protocol for an embedded mixed-methods process evaluation conducted alongside the HIPCARE cluster randomized controlled trial. The process evaluation aims to understand implementation, contextual factors, adoption, sustainability, and mechanisms underlying a multidisciplinary hip fracture care intervention across five low- and middle-income countries in Asia. Quantitative implementation metrics from participating hospitals will be integrated with qualitative interviews and focus groups involving clinicians, administrators, patients, and caregivers using RE-AIM and Realist evaluation frameworks.

Importance of the study

This protocol addresses an important question. Understanding barriers and facilitators to implementation of multidisciplinary hip fracture care in resource constrained settings has the potential to inform future scaling and sustainability efforts across LMICs.

General comments:

Timing of protocol publication

The manuscript states that process evaluation data will be collected throughout recruitment and follow up, yet the trial status section reports recruitment was scheduled to conclude on July 31, 2026. Given this timeline, it is unclear how much recruitment and process evaluation data collection had already been completed at the time of submission. The authors should clarify study status at submission, including the extent of recruitment, qualitative data collection, and whether any analyses had commenced, to help confirm compliance with the journal's policy that protocol manuscripts should describe planned or ongoing research and that protocols with completed data collection may not be considered for publication.

Selection bias

Only 10 of the intervention hospitals will participate in the process evaluation, but the manuscript does not clearly explain how these sites will be selected or what constitutes a representative sample. Additional detail is needed to assess the risk of selection bias.

Qualitative analysis

The planned combination of deductive coding based on realist principles followed by inductive theme generation is reasonable. However, additional details regarding coding reliability, management of translated transcripts, and resolution of coding disagreements would improve reproducibility.

Limitations

The authors should consider acknowledging additional limitations including cross-cultural variation in qualitative data collection and interpretation, potential information loss during transcription and translation, social desirability bias among implementation champions and hospital leadership, and challenges distinguishing intervention fidelity from local adaptation.

Lines 334-343

I found this section somewhat difficult to reconcile with the overall study rationale. The observation that several participating LMICs report lower mortality estimates than Australia and New Zealand appears to contrast with the earlier argument that limited adoption of multidisciplinary hip fracture care may contribute to poorer outcomes. However, this apparent paradox may actually strengthen the justification for the process evaluation by highlighting that the drivers of international outcome variation remain incompletely

understood. The authors could make this point more explicit and consider discussing other factors that may contribute to these differences.

Overall assessment

This is a well conceived and potentially valuable process evaluation embedded within a large multinational hip fracture trial. The theoretical framework, international collaboration, and implementation science focus are notable strengths. However, clarification regarding the timing of protocol publication relative to trial progress is essential, particularly the extent of recruitment and process evaluation activities completed at the time of submission. After clarifying these issues and addressing a few concerns with managing bias and clarity for reproducibility, the study has the potential to make a meaningful contribution to the implementation literature in hip fracture care.

VERSION 1 - AUTHOR RESPONSE

Table: Author responses to reviewer feedback

**REVIEWER #1 SPECIFIC COMMENTS**	
**Timing of protocol publication:** The manuscript states that process evaluation data will be collected throughout recruitment and follow up, yet the trial status section reports recruitment was scheduled to conclude on July 31, 2026. Given this timeline, it is unclear how much recruitment and process evaluation data collection had already been completed at the time of submission. The authors should clarify study status at submission, including the extent of recruitment, qualitative data collection, and whether any analyses had commenced, to help confirm compliance with the journal's policy that protocol manuscripts should describe planned or ongoing research and that protocols with completed data collection may not be considered for publication.	**Authors' response:** Thank you for emphasising the need to comply with the journal's policy on publishing protocols for planned or ongoing research. We understand the requirement for protocol submission prior to the end of recruitment and data collection to ensure compliance with BMJ Open policies. At the time of manuscript submission, the recruitment and data collection for the HIPCARE trial and process evaluation were ongoing. Data collection was scheduled to continue until the end of December 2026. We have revised the Introduction section of the manuscript to clarify that the research was ongoing and added additional information to the Methods and Analysis section.<br><br>**Manuscript locations: Page 8, lines 184–185**. *'…the planned recruitment period from November 2024 until 31 July 2026. Data collection and data quality checks for the main trial will continue until the end of December 2026.'*<br><br>**Page 11, lines 236–240**. *'Timing of process evaluation data collection in each country will be influenced by the date of the first patient recruitment to the main trial. Following the end of main trial recruitment on 31 July 2026, participant recruitment, data collection and*

	data quality checks for the process evaluation will continue until December 2026 for all five countries.'<br><br>**Page 11, line 249–250**. *'...and completion of recruitment on 31 July 2026 (endpoint).'*
**Selection bias:** Only 10 of the intervention hospitals will participate in the process evaluation, but the manuscript does not clearly explain how these sites will be selected or what constitutes a representative sample. Additional detail is needed to assess the risk of selection bias.	**Authors' response:** Thank you for this comment and we agree additional detail on site selection will provide greater clarity for the reader. We have added additional detail to the Methods and Analysis section of the manuscript, the Discussion section, and the Strengths and limitations dot points 2 and 3 on page 6.<br><br>**Manuscript location: Page 11, lines 240–244**. *'Ten of the 20 intervention hospitals will be purposively selected to ensure diversity across characteristics likely to influence implementation of team-based care, including hospital size, geographic location and service profile. This approach is designed to obtain a broad range of implementation contexts within each country and allow the capture of diverse implementation experiences. From a diverse, representative sample of 10 hospitals ...'*<br><br>**Page 17–18, lines 402–408.** '*Finally, as only a subset of intervention hospitals will participate in the process evaluation, experiences and contexts may not fully reflect non-participating sites. This may lead to variation in data generation and interpretation and may limit the transferability of identified barriers, enablers and adaptations; however, the process evaluation is designed to understand implementation within diverse, real-world settings and this may be considered a focus of the evaluation rather than a limitation of the proposed approach.'*
**Qualitative analysis:** The planned combination of deductive coding based on realist principles followed by inductive theme generation is reasonable. However, additional details regarding coding reliability, management of translated transcripts, and resolution of coding disagreements would improve reproducibility.	**Authors' response:** Thank you for this suggestion to improve reproducibility. We have provided additional detail regarding the coding procedures by revising the Methods section of the manuscript.<br><br>**Manuscript location: Page 14, lines 309–317.**

	'All transcripts will be independently coded by at least two researchers. Regular meetings will be held to compare coding, discuss interpretations, and refine the coding framework. Discrepancies in coding will be resolved through discussion and consensus with the involvement of researchers from each of the participating countries during the analysis process. For interviews conducted in languages other than English, audio files will be transcribed into the local language prior to translation into English-language by qualified translators or bilingual researchers. English-language transcripts will be reviewed for accuracy and original-language transcripts will be consulted during analysis when clarification of meaning is required.'
**Limitations:** The authors should consider acknowledging additional limitations including cross-cultural variation in qualitative data collection and interpretation, potential information loss during transcription and translation, social desirability bias among implementation champions and hospital leadership, and challenges distinguishing intervention fidelity from local adaptation.	**Authors' response:** Thank you for these thoughtful suggestions. We have considered each of the additional limitations proposed and have revised the Discussion section to acknowledge additional limitations.<br><br>**Manuscript location: Pages 17–18, lines 392–409**. *'With interview-based research there is the potential for bias due to cross-cultural differences in language, comprehension, emotions, experiences, professional norms, organisational cultures, or attitudes towards team-based care [18]. These differences may affect interpretation and comparability of the findings or country comparisons. There may also be some loss of nuance and contextual meaning during translation from local languages into English. Another limitation may be the potential for social desirability bias [19], whereby implementation champions, hospital leaders, and other stakeholders involved in intervention delivery provide socially desirable responses, potentially leading to more favourable descriptions of implementation processes and experiences. In addition, distinguishing between intervention fidelity and contextually appropriate adaptations may be challenging, particularly in complex health system settings where implementation strategies are intentionally tailored to local circumstances. Finally, as only a subset of intervention hospitals will participate in the process evaluation, experiences and contexts may not fully reflect non-participating sites. This may lead to variation in data generation*

	and interpretation and may limit the transferability of identified barriers, enablers and adaptations; however, the process evaluation is designed to understand implementation within diverse, real-world settings and this may be considered a focus of the evaluation rather than a limitation of the proposed approach.'
**Lines 334-343:** I found this section somewhat difficult to reconcile with the overall study rationale. The observation that several participating LMICs report lower mortality estimates than Australia and New Zealand appears to contrast with the earlier argument that limited adoption of multidisciplinary hip fracture care may contribute to poorer outcomes. However, this apparent paradox may actually strengthen the justification for the process evaluation by highlighting that the drivers of international outcome variation remain incompletely understood. The authors could make this point more explicit and consider discussing other factors that may contribute to these differences.	**Authors' response:** Thank you for this helpful observation. We agree that the apparent paradox of lower mortality in some LMICs despite limited implementation team-based care calls for further discussion. We have revised the Discussion section of the manuscript to reflect the complexity of factors that might be reflected by this inconsistency.<br><br>**Manuscript location: Page 16, lines 361–366**. *'Differences in patient case-mix, healthcare access and admission may contribute to the observed variation in survival following hip fracture. Understanding these variations across the diverse trial settings through the process evaluation may provide meaningful insights into the factors influencing the delivery (and subsequent outcomes) of hip fracture care. In this way, the process evaluation may contribute to the interpretation of country-level differences in observed patient outcomes.'*
**Overall assessment**: This is a well conceived and potentially valuable process evaluation embedded within a large multinational hip fracture trial. The theoretical framework, international collaboration, and implementation science focus are notable strengths. However, clarification regarding the timing of protocol publication relative to trial progress is essential, particularly the extent of recruitment and process evaluation activities completed at the time of submission. After clarifying these issues and addressing a few concerns with managing bias and clarity for reproducibility, the study has the potential to make a meaningful contribution to the implementation literature in hip fracture care.	**Authors' response:** Thank you for this comment. In the points addressed above, we have clarified the timing of protocol publication relative to trial progress and confirmed that the research was ongoing at the time of submission and believe we have addressed the identified concerns with managing bias and ensuring reproducibility. The manuscript is much improved by addressing the Reviewer's thoughtful comments and suggestions.<br><br>**Manuscript location:** N/A (see revisions addressing earlier points).
